# How to adjust the expected waiting time to improve patient’s satisfaction?

**DOI:** 10.1186/s12913-023-09385-9

**Published:** 2023-05-08

**Authors:** Hui Zhang, Wei-Min Ma, Jing-Jing Zhu, Li Wang, Zhen-Jie Guo, Xiang-Tang Chen

**Affiliations:** 1 School of Economics and Management, Tongji University, Shanghai, 200092 China; 2 Scientific Research Department, The First Affiliated Hospital of Zhejiang Chinese Medical University (Zhejiang Provincial Hospital of Traditional Chinese Medicine), Hangzhou, 310000 China; 3 Eye Hospital, Wenzhou Medical University at Hangzhou, Zhejiang Eye Hospital at Hangzhou, Hangzhou, 310000 China; 4 School of Economics and Management, Wenzhou University of Technology, Wenzhou, 325000 China

**Keywords:** Expected waiting time, Patient’s satisfaction, Actual waiting time

## Abstract

**Background:**

Long waiting time in hospital leads to patient’s low satisfaction. In addition to reducing the actual waiting time (AWT), we can also improve satisfaction by adjusting the expected waiting time (EWT). Then how much can the EWT be adjusted to attribute a higher satisfaction?

**Methods:**

This study was conducted though experimental with hypothetical scenarios. A total of 303 patients who were treated by the same doctor from August 2021 to April 2022 voluntarily participated in this study. The patients were randomly divided into six groups: a control group (*n* = 52) and five experimental groups (*n* = 245). In the control group, the patients were asked their satisfaction degree regarding a communicated EWT (T_0_) and AWT (T_a_) under a hypothetical situation. In the experimental groups, in addition to the same T_0_ and T_a_ as the control group, the patients were also asked about their satisfaction degree with the extended communicated EWT (T_1_). Patients in five experimental groups were given T_1_ values with 70, 80, 90, 100, and 110 min respectively. Patients in both control and experiment groups were asked to indicate their initial EWT, after given unfavorable information (UI) in a hypothetical situation, the experiment groups were asked to indicate their extended EWT. Each participant only participated in filling out one hypothetical scenario. 297 valid hypothetical scenarios were obtained from the 303 hypothetical scenarios given.

**Results:**

The experimental groups had significant differences between the initial indicated EWT and extended indicated EWT under the effect of UI (20 [10, 30] vs. 30 [10, 50], *Z* = -4.086, *P* < 0.001). There was no significant difference in gender, age, education level and hospital visit history (*χ*^2^ = 3.198, *P* = 0.270; *χ*^2^ = 2.177, *P* = 0.903; *χ*^2^ = 3.988, *P* = 0.678; *χ*^2^ = 3.979, *P* = 0.264) in extended indicated EWT. As for patient’s satisfaction, compared with the control group, significant differences were found when T_1_ = 80 min (*χ*^2^ = 13.511, *P* = 0.004), T_1_ = 90 min (*χ*^2^ = 12.207, *P* = 0.007) and T_1_ = 100 min (*χ*^2^ = 12.941, *P* = 0.005). When T_1_ = 90 min, which is equal to the T_a_, 69.4% (34/49) of the patients felt “very satisfied”, this proportion is not only significantly higher than that of the control group (34/ 49 vs. 19/52, *χ*^2^ = 10.916, *P* = 0.001), but also the highest among all groups. When T_1_ = 100 min (10 min longer than T_a_), 62.5% (30/48) of the patients felt “very satisfied”, it is significantly higher than that of the control group (30/ 48 vs. 19/52, *χ*^2^ = 6.732, *P* = 0.009). When T_1_ = 80 min (10 min shorter than T_a_), 64.8% (35/54) of the patients felt “satisfied”, it is significantly higher than that of the control group (35/ 54 vs. 17/52, *χ*^2^ = 10.938, *P* = 0.001). However, no significant difference was found when T_1_ = 70 min (*χ*^2^ = 7.747, *P* = 0.052) and T_1_ = 110 min (*χ*^2^ = 4.382, *P* = 0.223).

**Conclusions:**

Providing UI prompts can extend the EWT. When the extended EWT is closer to the AWT, the patient’s satisfaction level can be improved higher. Therefore, medical institutions can adjust the EWT of patient’s through UI release according to the AWT of hospitals to improve patient’s satisfaction.

**Supplementary Information:**

The online version contains supplementary material available at 10.1186/s12913-023-09385-9.

## Background

The experience of waiting in outpatient is usually the beginning of interaction between patients and hospitals [1]. So waiting time is regarded as one of the important indicators of service quality [2]. Because the demand for medical resources is greater than the supply, the problem of long waiting time in outpatient service is very common [3, 4]. Long waiting time can worsen patient’s health conditions [5], affect public confidence in health facilities [6] and reduce patient’s satisfaction [7–10].

Scholars and hospital managers have focused on efforts to lessen the actual waiting time (AWT) by improving the medical treatment process [11–23]. Some hospitals in China simplify the medical treatment process through online payment, which directly reduces the AWT of patients in the hospitals [15, 16]. Appointment is another way to reduce the AWT by matching the needs of patients with the supply of medical resources in time [17–20]. However, appointment no-show can reduce hospitals operational efficiency [21]. Hospital helps patients complete imaging examinations or laboratory tests before they consulting doctors through artificial intelligence technology to reduce the AWT [22]. They also try to reduce the AWT of patients by strengthening the personnel on duty during peak hours [23]. However, because patients in China prefer senior experts and advanced medical equipment, it leads to the short supply of high-quality medical resources and results in further dissatisfaction in large hospitals [24, 25].

In addition to focusing on the impact of objective factors such as the AWT and the hospital environment, scholars have paid attention to the psychological feelings of patients [26–28], because people’s evaluation or judgment of objective things is based on the reference point. Anything above the reference point is regarded as a gain, and people tend to give a positive evaluation when they gain something; by contrast, anything below the reference point is regarded as a loss, and people tend to give a negative evaluation when they lose something [29]. People’s evaluation of the same objective situation will change when the reference point changes [30]. Patients in the waiting area have a common feeling that they do not know how long they need to wait, and this uncertainty about waiting time will aggravate their dissatisfaction. In order to reduce uncertainty, it is proved that when individuals receive unfavorable information (UI), their expected waiting time (EWT) will be extended, and when the AWT is between the initial EWT and the extended EWT, the individuals are more satisfied than those who does not extend EWT [31]. This provides another direction besides focusing on the AWT for hospitals to improve patient’s satisfaction. Meanwhile, in the field of transportation, the AWT of passengers has been accurately predicted [32, 33]. This provides a possibility for the prediction of the AWT in outpatient. In fact, models that can be established to predict the needs of patients and the arriving time based on the previous outpatient data has been studied [34–36]. It is a step forward to predicting the AWT of patients.

Hence, in order to improve patient’s satisfaction and make the adjustment of the EWT can be more maneuverable, based on research before and around the AWT, this paper investigate how much can the EWT be adjusted to attribute a higher satisfaction. The main innovation is that we designed five different experimental groups with extended EWT around the AWT, and compared them with the control group that without the extended EWT one by one. And this experimental is conducted in the hospital and the experimental subjects are the patients.

## Methods

### Experiment design

It was found in healthy people that UI extended the EWT of subjects, and that, moreover, when the AWT was between the initial EWT and the extended EWT, patient’s satisfaction was improved significantly [31]. In order to investigate how much can the EWT be adjusted to attribute a higher satisfaction? By experiment, we designed a control group and five experimental groups with hypothetical scenarios. All the six groups of experiments consist of three parts: the first part is that patients indicate the EWT under the hypothetical situation. This part mainly explores the impact of UI on the EWT; in the second part, patients are asked to report satisfaction degree with communicated AWT and EWT in hypothetical scenarios. This part mainly discusses how much can the EWT be adjusted to attribute a higher satisfaction. The third part is the basic information of patients.

Specifically, in the first part of the experiments, patients in all groups were asked to indicate their initial EWT (T^*^_0_), and then, patients in experimental groups were asked to report their extended EWT (T^*^_1_) with the effect of UI in hypothetical scenarios. In the second part of the hypothetical scenario experiments, in the control group, the patients were asked to report their satisfaction of the initial communicated EWT (T_0_) and the communicated AWT (T_a_) under hypothetical scenarios (Table 1). In the experimental group scenarios, in addition to the same T_0_ and T_a_ as the control group, the patients were also asked about their satisfaction when the T_0_ was extended to the communicated EWT (T_1_). The difference between the five experimental groups was that patients were given different T_1_ values (experimental group 1: T_1_ = 70; experimental group 1: T_1_ = 80; experimental group 1: T_1_ = 90; experimental group 1: T_1_ = 100; experimental group 1: T_1_ = 110 min). In this way when T_0_ and T_a_ are fixed, patient’s satisfaction changing at different T_1_ can be intuitively analyzed. However, in actual situations, the T^*^_0_, T^*^_1_ and AWT of patients are different, this will make it difficult to distinguish between the AWT and the EWT when analyzing the satisfaction data. Gender, age, hospital experiences, and other basic information were also obtained.

**Table 1:** Experimental design of control group and experimental groups (minutes)

Group	T_a_	T_0_	T_1_
Control group 1	90	60	--
Experimental group 1	90	60	70
Experimental group 2	90	60	80
Experimental group 3	90	60	90
Experimental group 4	90	60	100
Experimental group 5	90	60	110

T_a_, the communicated actual waiting time; T_0_, initial communicated expected waiting time; T_1_, extended communicated expected waiting time.

“--” means no information.

In this study, the information related to the peak flow of patients was defined as UI. Satisfaction level was categorized as “very dissatisfied”, “dissatisfied”, “satisfied”, and “very satisfied”. The subjects were asked to choose one of the above four levels above to express their satisfaction degree.

### Subjects and setting

The study participants were patients who came to the hospital for treatment due to various vision problems. The reason for choosing such patients was to exclude the impact of objective factors on the evaluation results, such as physical pain and disease severity. All patients participating in the experiment were treated by the same doctor (doctor Guo) during the peak hours in every Monday morning from August 2021 to March 2022. In this way, the impact of different doctors’ medical skills and service attitudes on the evaluation results of patients can be weakened. The doctor we chose was a general expert and not a specialized expert, because the appointment of a specialized expert is relatively difficult for patients; then the psychological effect of compensation due to the appointment of a specialized expert would make the patients more willing to wait.

### Experimental implementation

The study was conducted in the outpatient department of Zhejiang Eye Hospital in Hangzhou from August 2021 to March 2022. The formula of sample size is expressed as follows: $n=\frac{{({t}_{1-\alpha }+{t}_{k})}^{2}{\sigma }^{2}}{{\beta }^{2}P(1-P)}$, where $n$ is the minimum sample size required for the experiment; with 5% significance level and 80% efficacy,${({t}_{1-\alpha }+{t}_{k})}^{2}={(1.96+0.84)}^{2}=7.84$;$P$ is the proportion of experimental group to total sample; $\beta$ is the intervention effect of experimental group compared to control group; $\sigma$ is the standard deviation [37]. Let $\beta =0.5\sigma$ [38], According to the formula, the minimum sample size is 226, expand the sample size by approximately 30% to carry out the survey. Firstly, we conducted professional training for the experiments’ organizer. Secondly, we randomly rank six groups of hypothetical scenarios, and patients on each Monday fill in one hypothetical scenario, and patients on the next Monday fill in another hypothetical scenario in random order. All hypothetical scenarios are available as supplementary information (Additional file 1). Thirdly, during peak hours every Monday morning, after the patients were registered, they went to the waiting room. Before the patients saw the doctor, the experiment organizer explained to them the purpose of the study and the efforts to protect privacy. Then, with the consent of the patients, the experiment organizer gave the hypothetical scenario to patients and collected them. A total of approximately 500 patients were approached, and 303 patients agreed to volunteer to participate in this experiment. Only 297 valid hypothetical scenarios, including 52 in the control group and 245 in the five experimental groups, were obtained. Finally, we transferred the data of the paper hypothetical scenario into the computer.

### Ethics statement

This experiment obtained the ethical permission of the Office of Research Ethics, Eye Hospital of Wenzhou Medical University, and each patient also signed an informed consent form when participating in the experiment. The study was conducted in accordance with the ethical principles of the Declaration of Helsinki. Participants were informed at the top of the hypothetical scenario about the purpose of the study and privacy protection. They were also told that they could quit at any time. The obtained data was analyzed anonymously.

### Variables

#### Demographic variables

The demographic variables included gender, age, hospital history, and education level.

#### Satisfaction level

Satisfaction level was categorized as “very dissatisfied”, “dissatisfied”, “satisfied”, and “very satisfied”.

### Hypothetical scenarios and tools

In the first part of the hypothetical scenarios, the control group was only asked to indicate their T^*^_0_. Experimental groups 1–5 were asked to indicate their T^*^_0_ and T^*^_1_. In the second part of the hypothetical scenarios, the control group were asked to report satisfaction evaluation with T_0_ and T_a_. The experimental groups 1–5 were given different extended T_1_ with 70, 80, 90, 100 and 110 min. In the third part of the experiment, all subjects were asked about their gender, age, education, and hospital visit experience.

The experiment organizer distributed paper hypothetical scenarios to the patients in the waiting room, and the patients filled in the hypothetical scenarios and returned them to the staff. Each patient completed the whole process within 8–10 min.

### Statistical analysis

Data analysis was performed on SPSS 22.0 and involved the non-parametric test of two independent samples. P *<* 0.05 indicates a significant difference between the two sample data. We also used descriptive statistical methods.

## Results

### Baseline characteristics

A total of 303 patients participated in the experiment, and 297 valid hypothetical scenarios were obtained. The proportion of valid hypothetical scenarios was 98.0%, including 52 cases in the control group and 245 cases in the experimental groups. The experimental and control groups had 155 (63.3%) and 27 (51.9%) females respectively (Table 2). No significant differences in gender composition ratios were observed (*P* > 0.05). The median age of the subjects was 35.5 years old in the control group and 37.0 years old in the experimental group. The two groups had no significant differences in median age (*P* > 0.05). In the control group, 57.7% of the subjects received graduate education, which was equivalent to the proportion of subjects with the same education level in the experimental groups (*P* > 0.05). Most subjects (78.8%) had a history of hospital visits.

**Table 2:** Baseline characteristics of the study subjects

Group	Female	Age (years old)	Graduate	Hospital visit history
Control group (n = 52)	27 (51.9%)	35.5 [29.0, 40.0]	30 (57.7%)	41 (78.8%)
Experimental group (n = 245)	155 (63.3%)	37.0 [34.0, 40.0]	142 (58.0%)	193 (78.8%)
Experimental group 1 (n = 45)	20 (44.4%)	38.0 [33.0, 42.0]	27 (60.0%)	31 (68.9%)
Experimental group 2 (n = 54)	35 (64.8%)	36.0 [33.5, 40.0]	32 (59.3%)	45 (83.3%)
Experimental group 3 (n = 49)	34 (69.4%)	36.0 [30.0, 40.0]	25 (51.0%)	42 (85.7%)
Experimental group 4 (n = 48)	33 (68.8%)	37.5 [35.0, 39.75]	29 (60.4%)	34 (70.8%)
Experimental group 5 (n = 49)	33 (67.3%)	37.0 [34.0, 40.0]	29 (59.2%)	41 (83.7%)

In the five experiment groups, there were no significant differences in gender, age, education and hospital visit history (*χ*^*2*^ = 8.642, *P* = 0.071; *χ*^*2*^ = 3.284, *P* = 0.512; *χ*^*2*^ = 0.250, *P* = 0.993; *χ*^*2*^ = 8.371, *P* = 0.079).

### Unfavorable information extends EWT

In the experimental groups, the median of T^*^_0_ was 20 min before UI was given, 57.1% (140/245) of the patients extended their EWT after UI was given, and the median of patient’s T^*^_1_ was 30 min. patient’s T^*^_0_ and T^*^_1_ in the experimental groups had a significant difference (20 [10, 30] vs. 30 [10, 50], *Z* = − 4.086, *P* < 0.001). There were no significant differences of the patient’s T^*^_1_ in gender (*χ*^2^ = 3.198, *P* = 0.270), age (*χ*^2^ = 2.177, *P* = 0.903), or education levels (*χ*^2^ = 3.988, *P* = 0.678). Moreover, the patient’s T^*^_1_ between patients with and without a hospital visit history was similar (*χ*^2^ = 3.979, *P* = 0.264). Ratio of the patients extended the EWT by 1–20 min is 35.9%, and ratio of the patients extended their EWT by 41 min or more is 3.7% (Table 3).

**Table 3:** Extension of expected waiting time (EWT) in the experimental groups

	N	≤ 0 min	1–20 min	21–40 min	≥ 41 min
All subjects	245	105(42.6%)	88(35.9%)	43(17.6%)	9(3.7%)
Gender					
Male	90	40(44.4%)	30(33.3%)	19(21.1%)	1(1.1%)
Female	155	65(41.9%)	58(37.4%)	24(15.5%)	8(5.2%)
Hospital visit history					
Yes	193	83(43.0%)	73(37.8%)	29(15.0%)	8(4.1%)
No	46	20(43.5%)	13(28.3%)	12(26.1%)	1(2.2%)
Education level					
High school and below	71	34(47.9%)	26(36.6%)	9(12.7%)	2(2.8%)
Graduate	142	58(40.8%)	49(34.5%)	29(20.4%)	6(4.2%)
Post Graduate	30	13(43.3%)	13(43.3%)	3(10%)	1(3.3%)
Age					
18 years old and under	37	16(43.2%)	15(40.5%)	5(13.5%)	1(2.7%)
19–36 years old	77	35(45.5%)	23(29.9%)	16(20.8%)	3(3.9%)
37 years old and above	131	55(42.0%)	49(37.4%)	22(16.8%)	5(3.8%)

### The extended EWT closer to AWT attribute a higher satisfaction

Patient’s satisfaction was compared between the control and experimental groups. All groups were given the same T_a_ and T_0_ as shown in Table 4. Significant differences in patient’s satisfaction were found compared with the control group when T_1_ = 80 min (*χ*^2^ = 13.511, *P* = 0.004), T_1_ = 90 min (*χ*^2^ = 12.207, *P* = 0.007), and T_1_ = 100 min (*χ*^2^ = 12.941, *P* = 0.005). However, no significant differences in patient’s satisfaction were found when T_1_ = 70 min (*χ*^2^ = 7.747, *P* = 0.052) and T_1_ = 110 min (*χ*^2^ = 4.382, *P* = 0.223) compared with the control group. This result is basically consistent with research before [31].

When T_1_ = 80 min, 64.8% (35/54) of the patients felt “satisfied” after a longer T_1_ was given (Table 4). This ratio was higher than that of the control group (35/54 vs. 17/52, *χ*^2^ = 10.938, *P* = 0.001). Moreover, the patients who felt “dissatisfied” significantly decreased (5/54 vs. 13/52, *χ*^2^ = 4.656, *P* = 0.031). When T_1_ = 90 min, 69.4% (34/49) of the patients felt “very satisfied”. This proportion was significantly higher than that of the control group (34/49 vs. 19/52, *χ*^2^ = 10.916, *P* = 0.001) and it was the highest among all groups. When T_1_ = 100 min, the individuals who felt “dissatisfied” significantly decreased (1/48 vs. 13/52, *χ*^2^ = 10.887, *P* = 0.001), however the patients who felt “very satisfied” increased significantly (30/48 vs. 19/52, *χ*^2^ = 6.732, *P* = 0.009).

**Table 4:** Satisfaction in the control and experimental groups

Group	Cases	Very dissatisfied	Dissatisfied	Satisfied	Very satisfied
Control group					
T_0_ = 60, T_a_ = 90	52	3 (5.8%)	13 (25.0%)	17 (32.7%)	19 (36.5%)
Experimental groups					
T_0_ = 60, T_1_ = 70 T_a_ = 90	45	0 (0.0%)	4 (8.9%)	21 (46.7%)	20 (44.4%)
T_0_ = 60, T_1_ = 80, T_a_ = 90	54	0 (0.0%)	5 (9.3%) ^a^	35 (64.8%) ^a^	14 (25.9%)
T_0_ = 60, T_1_ = 90, T_a_ = 90	49	0 (0.0%)	6 (12.2%)	9 (18.4%)	34 (69.4%) ^a^
T_0_ = 60, T_1_ = 100, T_a_ = 90	48	2 (4.2%)	1 (2.1%) ^a^	15 (31.3%)	30 (62.5%) ^a^
T_0_ = 60, T_1_ = 110, T_a_ = 90	49	5 (10.2%)	5 (10.2%)	16 (32.7%)	23 (46.9%)

^a^Compared to control group, *P* < 0.05.

## Discussion

The disadvantages of waiting for a long time are obvious, such as increased hospital costs [39] and decreased patient’s satisfaction. In China, many hospitals have implemented online appointment and mobile payment, which has partially alleviated the overcrowding and improved patient’s satisfaction in recent years [40]. But the tertiary hospitals have a large number of critically ill patients from rural areas; compared with limited medical resources, waiting for a long time in the tertiary hospitals is still a prominent problem in China [24].

The initial EWT of the patients is usually based on the previous experience. After patients arrive at the hospital, their EWT will be affected by the medical conditions observed by the patient and the information provided by the hospital. The EWT of patients can be considerably prolonged under the influence of UI. This finding is consistent with the result of a previous experiment [31]. Individuals tend to be more alert to UI to eliminate potential hazards [41]. By doing so, their sense of loss will not make them particularly uncomfortable when their expectations are adjusted to be closer to the final actual situation. However, we also noticed that 42.6% of the patients in the experimental groups did not extend their EWT after receiving UI about the waiting time. We speculated that it might be because there are fewer people in the waiting area than they expected; therefore, they adjusted the EWT according to the actual situation on the spot rather than the hypothetical scenario given in the experiment.

Patient’s satisfaction was improved by extending the EWT. For the same AWT, the experimental groups had higher satisfaction levels compared with the control group. The difference was that the experimental groups received UI and adjusted the EWT. The EWT can be regarded as a reference point [31], and the reference point is the core factor determining people’s evaluation results [42]. People will depend on the reference point, which means that individuals make decisions based on gains and losses relative to the reference point and not only on the actual result [43]. If the actual situation is better than the reference point, then individuals tend to give a positive evaluation; otherwise, they tend to give a negative evaluation [44].

However, the adjustment of the EWT caused by UI prompts provided cannot substantially improve patient’s satisfaction in all cases. It was found that when the AWT was between the initial EWT and the extended EWT under the influence of UI, the patient’s satisfaction was significantly improved [31]. This shows that the impact of extended EWT on patient’s satisfaction is limited. On this basis, this study further investigated how much can the EWT be adjusted to attribute a higher satisfaction, we found that patient’s satisfaction was improved when the EWT was adjusted closer to the AWT. Because uncertainty is one of the most significant factors leading to anxiety and other negative emotions [45]. Information about the waiting time can reduce the uncertainty of the waiting time and reduce the pressure on patients. Moreover, accurate information can enhance the patient’s trust in the hospital service level and thus increase patient’s satisfaction. However, patient’s satisfaction does not improve when the adjusted EWT is far from the AWT. It was probably because the patients felt cheated when they found that the AWT was very different from the information released by the hospital.

## Limitations

The main limitation of this study is that this experiment was carried out only in the consulting room of a doctor in an optometry hospital, and most of the patients have only slight physiological discomfort. Therefore, the tolerance of waiting time may be different from those with other types of diseases and physiological pain. Therefore, patient’s tolerance to the AWT under different degrees of physiological discomfort and the satisfaction with the adjustment of the EWT are topics worthy of further discussion.

## Conclusions

Through the behavioral experiment in the hospital, we conclude that the release of UI based on the actual medical situation in the hospital can extend patient’s EWT. An extended EWT closer to the AWT results in a higher overall satisfaction level. It helps the adjustment of the EWT more targeted. The method of improving patient’s satisfaction through real-time information release is applicable to hospitals with a serious queuing problem. Therefore, medical institutions can also pay attention to the EWT of patients while reducing the AWT, because this economical method can also help improve patient’s satisfaction and more practical for hospital management.

## Electronic supplementary material

Below is the link to the electronic supplementary material.

Additional file 1

## Data Availability

The datasets used and/or analyzed during the current study are available from the corresponding author on reasonable request.

## Abbreviations

AWT: The actual waiting time
EWT: The expected waiting time
T^*^_0_: The initial indicated expected waiting time
T^*^_1_: The extended indicated expected waiting time
T_0_: The initial communicated expected waiting time
T_a_: The extended communicated actual waiting time
T_1_: The extended communicated expected waiting time

## References

[1] Davis M, Heineke J (1998). How disconfirmation, perception and actual waiting times impact customer satisfaction. Int J Serv Ind Manag.

[2] Cao W, Wan Y, Tu H, Shang F, Liu D, Tan Z (2011). A web-based appointment system to reduce waiting for outpatients: a retrospective study. BMC Health Serv Res.

[3] Li JL, Zhu GJ, Luo L et al. Big Data-Enabled Analysis of Factors Affecting Patient Waiting Time in the Nephrology Department of a Large Tertiary Hospital. J Healthc Eng.2021; 2021:1–10. 10.1155/2021/5555029 PMC8178001 34136109

[4] Mahmoudzadeh H, Shalamzari AM, Abouee-Mehrizi H (2020). Robust multi-class multi-period patient scheduling with wait time targets. Oper Res Health Care.

[5] Reichert A, Jacobs R (2018). The impact of waiting time on patient outcomes: evidence from early intervention in psychosis services in England. Eur J Health Econ.

[6] Hosseininejad SM, Aminiahidashti H, Pashaei SM (2017). Determinants of prolonged length of stay in the emergency department: a cross-sectional study. Scand J Trauma Resus.

[7] Delucia PR, Mork KS, Ott TE et al. Measurement of the relationship between patient wait time and patient satisfaction at each stage of an appointment. Proceedings of the Human Factors & Ergonomics Society Annual Meeting. 2007; 51(18):1277-9.

[8] Luo J, Liu P, Wong M (2018). Patients’ satisfaction with dental care: a qualitative study to develop a satisfaction instrument. BMC Oral Health.

[9] Bleustein C, Rothschild DB, Valen A (2014). Wait times, patient satisfaction scores, and the perception of care. Am J Manag Care.

[10] Löflath V, Hau EM, Garcia D (2021). Parental satisfaction with waiting time in a swiss tertiary pediatric emergency department. Emerg Med J.

[11] Spaite DW, Bartholomeaux F, Guisto J (2002). Rapid process redesign in a university-based emergency department: decreasing waiting time intervals and improving patient satisfaction. Ann Emerg Med.

[12] Munavalli JR, Rao SV, Srinivasan A (2020). Integral patient scheduling in outpatient clinics under demand uncertainty to minimize patient waiting times. Health Inf J.

[13] Huang YL, DeisherAJ, Herman MG (2021). Reduce patient treatment wait time in a Proton Beam Facility - A Gatekeeper Approach. J Med Syst.

[14] Zhou ML, Loke GG, Bandi C et al. Intraday Scheduling with Patient Re-entries and Variability in Behaviours. M&SOM-Manuf Serv Op. 2022; 24 (1):561–579.

[15] Shen J, Zhang J, He Q (2021). Without the need for a second visit” initiative improves patient satisfaction with updated services of outpatient clinics in China. BMC Health Serv Res.

[16] Xie W, Yang X, Cao X, Liu P (2019). Effects of a comprehensive reservation service for non-emergency registration on appointment registration rate, patient waiting time, patient satisfaction and outpatient volume in a tertiary hospital in China. BMC Health Serv Res.

[17] Steenland M, Dula J, de Albuquerque A (2019). Effects of appointment scheduling on waiting time and utilization of antenatal care in Mozambique. BMJ Global Health.

[18] Ye Q, Wu H (2022). Patient’s decision and experience in the multi-channel appointment context: an empirical study. Front Public Health.

[19] Kong QX, Li S, Liu N (2020). .Appointment Scheduling under Time-Dependent Patient No-Show Behavior. Manag Res.

[20] Ali A, Chen F. Appointment scheduling problem in complexity systems of the healthcare services: A comprehensive review. J Healthc Eng. 2022; 2022:1–16. 10.1155/2022/5819813 PMC8913063 35281532

[21] Rosenbaum JI, Mieloszyk RJ, Hall CS, Hippe DS (2018). Understanding why patients no-show: observations of 2.9 million outpatient imaging visits over 16 years. J Am Coll Radiol.

[22] Li X, Tian D, Li W (2021). Artificial intelligence-assisted reduction in patient’s waiting time for outpatient process: a retrospective cohort study. BMC Health Serv Res.

[23] Sun J, Lin Q, Zhao P (2017). Reducing waiting time and raising outpatient satisfaction in a chinese public tertiary general hospital-an interrupted time series study. BMC Public Health.

[24] Yan J, Yao J, Zhao D (2021). Patient satisfaction with outpatient care in China: a comparison of public secondary and tertiary hospitals. Int J Qual Health C.

[25] Ren WC, Sun L, Tarimo CL (2021). The situation and infuencing factors of outpatient satisfaction in large hospitals: evidence from Henan province, China. BMC Health Ser Res.

[26] Ogunfowokan O, Mora M (2012). Time, expectation and satisfaction: patients’ experience at National Hospital Abuja, Nigeria. Afr J Prim Health Care Family Med.

[27] Soremekun OA, Takayesu JK, Bohan SJ (2011). Framework for analyzing wait times and other factors that impact patient satisfaction in the emergency department. J Emerg Med.

[28] Batbaatar E, Dorjdarva J, Lusannyam A (2016). Determinants of patient satisfaction: a systematic review. Perspect Public Health.

[29] Cheng M, Frangopol DM (2022). Life-cycle optimization of structural systems based on cumulative prospect theory: effects of the reference point and risk attitudes. Reliab Eng Syst Safe.

[30] Arkes HR, Hirshleifer D, Jiang D, Lim S (2008). Reference point adaptation: tests in the domain of security trading. Organ Behav Hum Dec.

[31] Ma WM, Zhang H, Wang NL (2019). Improving outpatient satisfaction by extending expected waiting time. BMC Health Serv Res.

[32] Sankaranarayanan HB, Agarwal G, Rathod V. An Exploratory Data Analysis of Airport Wait times Using Big Data Visualisation Techniques. 2016 International Conference on Computation System and Information Technology for Sustainable Solutions (CSITSS). Bengaluru, India, 2016; 324–329.

[33] Williams BM, Hoel LA. Modeling and forecasting vehicular traffic flow as a seasonal ARIMA process: Theoretical basis and empirical results: Proceedings of the American Society of Civil Engineers. J Transp Eng. 2003; 129(6):664–672.

[34] Bussel EV, van der Voort MBVR, Wessel RN (2018). Demand, capacity, and access of the outpatient clinic: a framework for analysis and improvement. J Eval Clin Pract.

[35] Klute B, Homb A, Chen W (2019). Predicting Outpatient appointment demand using machine learning and traditional methods. J Med Syst.

[36] Munavalli JR, Rao SV, Srinivasan A (2017). A robust Predictive Resource Planning under demand uncertainty to improve Waiting Times in Outpatient clinics. J Health Manag.

[37] Lu FW (2017). Random Field experiments: methods, Trends, and prospects. Economic Rev.

[38] Duflo E, Glennerster R, Kremer MR (2007). Using randomization in Development Economics Research: a Toolkit. Handb Dev Econ.

[39] Van Essen D, Vergouwen M, Sayre EC (2022). Does waiting times decrease or increase operational costs in short and long-term? Evidence from portuguese public hospitals. Injury.

[40] Zhou XJ, He QW, Li Q (2022). Factors Associated with Outpatient satisfaction in Provincial Tertiary Hospitals in Nanchang, China: a structural equation modeling Approach. Int J Env Res Pub He.

[41] Nam Y, Park HG, Kim YH (2022). Do you favor positive information or dislike negative information? Cultural variations in the derivation of the framing effect. Curr Psychol.

[42] Werner KM, Zank H (2019). A revealed reference point for Prospect Theory. Econ Theor.

[43] Schmidt U, Zank H (2012). A genuine foundation for prospect theory. J Risk Uncertain.

[44] Ma WM, Zhang H, Sun BZ et al. Dynamic hybrid multiple attribute decision-making problem based on reference point adaptation. Mathematical Problems in Engineering. 2019; 2019 (2019):1–9.

[45] Li J, Xia Y, Cheng XY (2020). Fear of uncertainty makes you more anxious? Effect of intolerance of uncertainty on college students’ social anxiety: a moderated mediation model. Front Psychol.

