# Supplementary material for: How to adjust the expected waiting time to improve patient’s satisfaction?
Source: BMC Health Serv Res. 2023 May 8;23:455. doi: 10.1186/s12913-023-09385-9 (PMC10169334; doi:10.1186/s12913-023-09385-9)
Supplement: Supplementary file 1 — Additional file 1 [file 12913_2023_9385_MOESM1_ESM.docx]

**Control Group**

Thank you very much for your willingness to participate in this questionnaire. Please complete the questionnaire according to the actual situation. This questionnaire is only for academic research. There is no right or wrong answer. Thank you for your cooperation. The results of the study will help hospitals to carry out outpatient work better. Waiting time refers to the time from registration to entering the consulting room. You can stop answering questions at any time. Next, please read carefully and answer the following questions:

Q1: How long do you expect to wait today before you come to the hospital? ________

Q2: If you expect to wait 60 minutes, but you actually wait 90 minutes. Only evaluate the waiting time, please give a satisfaction score.________

(0-25 points: very dissatisfied; 26-50 points: dissatisfied; 51-75 points: satisfied; 76-100 points: very satisfied)

Q3: How do you register today?

○Appointment ○Onsite registration

Q4: Your age: _____

Q5: Your education level

○high school and below ○Graduate ○postgraduate

Q6: Your gender

○ Male ○ Female

Q7: Have you ever been to the hospital before (including accompanying others)

○ Yes ○ No

**Experimental group 1-5**

Thank you very much for your willingness to participate in this questionnaire. Please complete the questionnaire according to the actual situation. This questionnaire is only for academic research. There is no right or wrong answer. Thank you for your cooperation. The results of the study will help hospitals to carry out outpatient work better. Waiting time refers to the time from registration to entering the consulting room. You can stop answering questions at any time. Next, please read carefully and answer the following questions:

Q1: How long do you expect to wait today before you come to the hospital? ________

Q2: When you get the information that there are many patients today, then how long do you expect to wait today? ______

Q3: If you expect to wait 60 minutes, you get the information that there are many patients today then your expected waiting time was adjusted to 70 (80, 90, 100, 110) minutes. While you actually waited 90 minutes.

Please give your comments on today's waiting time:_______________.

(0-25 points: very dissatisfied; 26-50 points: dissatisfied; 51-75 points: satisfied; 76-100 points: very satisfied. )

Q4: How do you register today?

○Appointment ○Onsite registration

Q5: Your age: _____

Q6: Your education level

○High school and below ○Graduate ○Postgraduate

Q7: Your gender

○ Male ○ Female

Q8: Have you ever been to the hospital before (including accompanying others)

○ Yes ○ No
